# Changes in Balance, Gait and Electroencephalography Oscillations after Robot-Assisted Gait Training: An Exploratory Study in People with Chronic Stroke

**DOI:** 10.3390/brainsci10110821

**Published:** 2020-11-06

**Authors:** Hoon-Ming Heng, Ming-Kuei Lu, Li-Wei Chou, Nai-Hsin Meng, Hui-Chun Huang, Masashi Hamada, Chon-Haw Tsai, Jui-Cheng Chen

**Affiliations:** 1 Neuroscience Laboratory, Department of Neurology, China Medical University Hospital, Taichung City 404, Taiwan; blacksun_kid@hotmail.com (H.-M.H.); d4297@mail.cmuh.org.tw (M.-K.L.); D12483@mail.cmuh.org.tw (H.-C.H.); D8079@mail.cmuh.org.tw (C.-H.T.); 2 School of Medicine, College of Medicine, China Medical University, Taichung City 404, Taiwan; 3 Department of Rehabilitation, Asia University Hospital, Taichung City 404, Taiwan; chouliwe@mail.cmuh.org.tw; 4 Department of Physical Medicine and Rehabilitation, China Medical University Hospital, Taichung City 404, Taiwan; D6351@mail.cmuh.org.tw; 5 Department of Physical Therapy and Graduate Institute of Rehabilitation Science, China Medical University, Taichung City 404, Taiwan; 6 Department of Neurology, The University of Tokyo, Graduate School of Medicine, Tokyo 100-0000, Japan; mhamada@m.u-tokyo.ac.jp; 7 Department of Neurology, China Medical University Hsinchu Hospital, Hsinchu 300, Taiwan

**Keywords:** ERD/ERS, RAGT, stroke, balance, gait

## Abstract

Robot-assisted gait training (RAGT) systems offer the advantages of standard rehabilitation and provide precise and quantifiable control of therapy. We examined the clinical outcome of RAGT and analyzed the correlations between gait analysis data and event-related desynchronization (ERD) and event-related synchronization (ERS) in patients with chronic stroke. We applied the Berg balance scale (BBS) and analyzed gait parameters and the ERD and ERS of self-paced voluntary leg movements performed by patients with chronic stroke before and after undergoing RAGT. A significant change was observed in BBS (*p* = 0.011). We also showed preliminary outcomes of changes in gait cycle duration (*p* = 0.015) and in ipsilesional ERS in the low-beta (*p* = 0.033) and high-beta (*p* = 0.034) frequency bands before and after RAGT. In addition, correlations were observed between BBS and ipsilesional ERS in the alpha and low-beta bands (*r* = −0.52, *p* = 0.039; *r* = −0.52, *p* = 0.040). The study demonstrated that RAGT can improve balance and provided an idea of the possible role of brain oscillation and clinical outcomes in affecting stroke rehabilitation.

## 1. Introduction

Motor function recovery after stroke is related to changes in the plasticity of the motor cortex and related motor areas. These altered activation patterns can revert to their original state, with normalization of reduced excitability and increase in the size of the cortical representation of motor function. Changes in plasticity have been a topic of interest in many neuroscience and neurorehabilitation studies [1,2,3,4,5].

Motor function of the lower extremities mainly concerns balance and gait and is the foundation for performing activities in daily life [6]. Balance and gait have been reported to be correlated [7]. After stroke, walking and balance abilities typically decline [8]. The Berg balance scale (BBS) is an assessment of balance with high inter-rater and interrater reliability, especially for people who have had a stroke [9]. It also has strong validity for inpatients with stroke [10]. Moreover, gait analysis has been suggested for use in assessing and improving walking ability in patients with stroke [11]. Gait analysis can be used as a parameter of motor function (walking ability) of the lower extremities. Thus, both the BBS and gait analysis can be employed for evaluating balance and gait, which can serve as parameters for assessing improvements in motor function of the lower extremities after a rehabilitation intervention.

With the greying demographics, health care is under great pressure. Robotics can provide more rehabilitation services to save manual labor from therapists. Robot-assisted gait training (RAGT) is an approach for training the lower extremities [12], that can also provide precise and quantifiable control of therapy, allowing better research into treatment dosage [13]. Several studies have reported the clinical efficacy of RAGT in enhancing balance and walking abilities [14,15,16]. Besides, studies are beginning to focus on the underlying mechanisms of RAGT. The relationship between alterations in gait and brain activation in patients after stroke has been explored in functional magnetic resonance imaging research [17]. Research with diffusion tensor imaging (DTI) also indicated that RAGT can facilitate plasticity in the intact supplementary motor area in the affected hemisphere [18]. However, it was inconclusive about the details regarding changes in the plasticity of the brain after RAGT and the relationship between cortical activation and motor function of the lower extremities. Understanding changes in the plasticity of the brain after improvements in balance and gait among patients with stroke is crucial and can reveal possible brain mechanisms underlying the efficacy of RAGT interventions.

Motor-related changes in electroencephalography (EEG) oscillation, such as event-related desynchronization (ERD), reflect phasic changes in limb movement associated with the synchrony of cell populations [19], and are possible markers of increased neuronal excitability in thalamocortical systems. Movement-related beta desynchronization, which is caused by electrophysiological signals from EEG or magnetoencephalography (MEG) in the contralateral primary cortex, was reported to be impaired in patients with stroke compared with healthy controls [3]. EEG analysis was first applied in healthy individuals during RAGT and mu and beta rhythms were found to be suppressed in central midline areas during active compared to passive walking [20]. It was also suggested to be possibly related to functional recovery after rehabilitation [2]. Besides, related neuromagnetic imaging research suggested that cortical excitation may be related to balance ability [21,22]. EEG was also found to be correlated to clinical improvement after gait training [23]. To this end, EEG enables the investigation of functional brain recovery across the cortico-basal network as well as movement related sensory interferences in the sensorimotor network [24,25,26]. ERD analysis has been employed in research on robot-assisted hand performance [27]. Changes in balance ability may be reflected in EEG oscillation changes.

In this study, we investigated BBS scores to support the clinical efficacy of RAGT and enhancing reproducibility. We also explored changes in the plasticity of the brain after RAGT by analyzing ERD, event-related synchronization (ERS). In addition, we have been the first to study whether motor-related neural oscillation and BBS scores are correlated.

## 2. Materials and Methods

### 2.1. Participants

Twenty-four patients with chronic stroke were recruited at clinics and consented. We specified that there were no consequences for participating or not participating in the experiment and that the participants were free to withdraw from the experiment at any time. After participants provided consent, they were screened to determine whether they met the inclusion and exclusion criteria. The inclusion criteria were as follows: (1) Between the ages of 35 and 80 years, (2) first diagnosis of a single unilateral subcortical stroke, as verified through brain imaging, (3) functional disability of a lower limb, and (4) ability to comprehend instructions for study participation (5) the onset of stroke more than 3 months. The exclusion criteria were as follows: (1) Deemed by a physician to be medically unstable, (2) presentation of cognitive impairments that would hinder safe participation in the study (Mini-Mental State Examination <23), (3) other prior musculoskeletal conditions that affect gait capacity, and (4) coexistence of other neurological diseases. Only participants who met all of the inclusion criteria and none of the exclusion criteria were recruited. We also recruited age-matched healthy controls to obtain age-matched parameter values. The experiments were confirmed to meet the standards set by the Declaration of Helsinki and were performed with the approval of the China Medical University Research Ethics Committee (Taichung City, Taiwan) (Ethic approval code: CMUH105-REC2-048; Date: 12 June 2016). All participants signed written informed consent forms.

#### 2.1.1. Protocol

**Figure 1:**
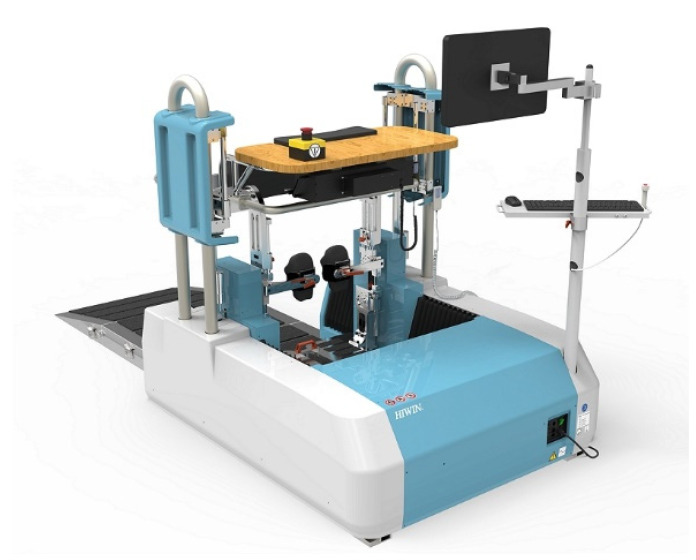
A photo of the gait training system.

Twenty-four patients with chronic stroke were recruited from the Department of Physical Medicine and Rehabilitation or Department of Neurology at China Medical University Hospital and randomized into two groups with 12 patients each, one for traditional rehabilitation and one for RAGT. A photo of the gait training system is shown in Figure 1. Participants in the traditional rehabilitation group received standard hospital-based rehabilitation management for stroke, which consisted of three sessions (30–45 min each) per week over the course of 4 weeks. Those in the RAGT group received the same standard hospital rehabilitation treatment along with an additional 30 min of RAGT with the MRG-P100 HIWIN Robotic Gait Training System after each of their session. For the RAGT program, an experienced physical therapist first assisted in transferring the patient to the robotic device by using a built-in patient transfer system. The therapist then followed and monitored the patients throughout the training period. On the basis of each patient’s comfort level and vital signs, the therapist adjusted the walking speed in real time while patients walked continuously from level 1 (slowest) to 10 (fastest).

#### 2.1.2. Primary Clinical Scores

**Figure 2:**
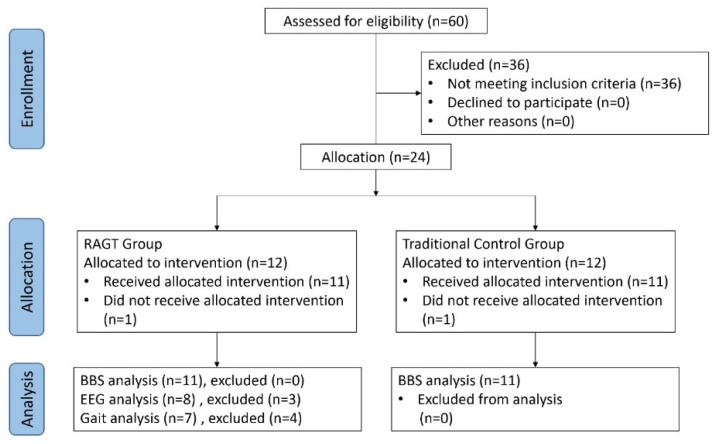
Flow chart of the study sample and examinations of the robot-assisted gait training (RAGT) and traditional treatment groups.

We performed The Berg balance scale (BBS) assessment before and after the 4-week rehabilitation program for each patient (Figure 2). The evaluator was blinded to the patients’ training assignment.

### 2.2. Secondary Parameter Measurements

#### 2.2.1. EEG and EMG Recordings

**Figure 3:**
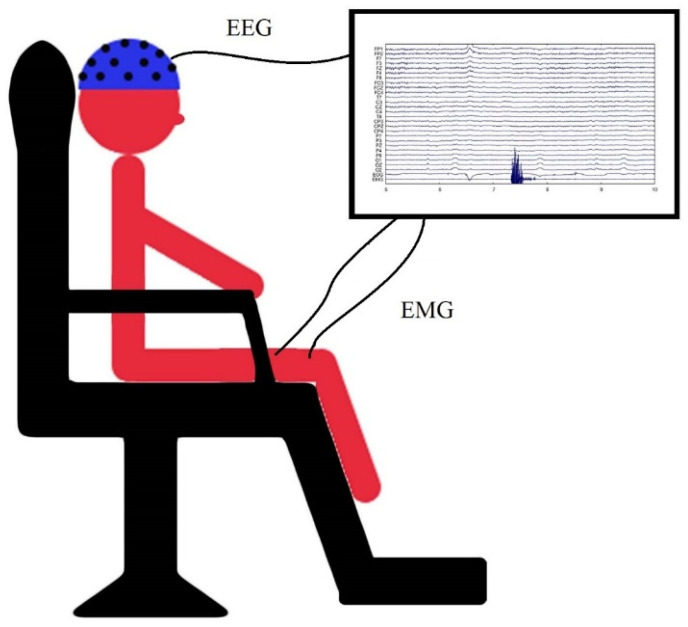
A visual illustration of lab setup for the electroencephalography (EEG) recording.

A visual illustration of lab setup for the EEG recording was showed in Figure 3. Patients were seated comfortably in an armchair with the affected leg and foot placed flat on the footrest. They performed knee extension–relaxation movements in the affected leg. In this tonic movement, patients extended the knee and sustained the posture for 7 s before returning the leg back to its original flat position. The time interval between each consecutive movement was approximately 7 s; four experimental blocks were conducted, with each block lasting for 4 min with a 1 min break in between. Patients were asked to focus on a red button in front of their eyes at a distance of 1.5 m while performing the movement. EEG data were acquired using a cap (Neuroscan) with 27 Ag/AgCl electrodes positioned according to the 10–20 system [28], (impedance was maintained at less than 5 kΩ), and two surface electrodes were placed on the vastus intermedius muscle to receive electromyography (EMG) signals. The reference point was established anterior to Fz, and the ground point was established posterior to Pz. EEG and EMG data were acquired at a rate of 1000 Hz with Neuroscan EEG System software and analyzed using MATLAB. Of the 11 patients in the RAGT group, eight were eligible for EEG recording and three were unable to complete the self-paced knee extension task and thus withdrew from EEG recording. Age-matched healthy controls also participated in EEG recording.

#### 2.2.2. ERD/ERS Analyses

During preprocessing, artifacts in the EEG recordings (i.e., eye movements, cardiac activity, and scalp muscle contraction) were removed with an independent component analysis procedure. The data were processed offline with an average reference and band-pass filter from 1 to 30 Hz. The EMG signals were rectified and normalized to identify the onset and offset of movement. The signals were then marked using a threshold of 1 for the absolute z-score. The EEG data were divided into epochs according to the onset and offset markers of the EMG signals.

**Figure 4:**
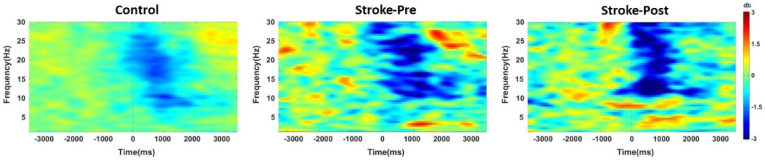
Illustration of the average event-related spectral perturbation at movement onset (0 m/s) for the healthy controls (left) and patients with stroke before RAGT (middle) and after RAGT (right). Clear event-related desynchronization (ERD) (blue color) followed by event-related synchronization (ERS) (red color) was observed in both the control and RAGT groups between the alpha and beta ranges in ipsilesional C3 equivalent electrode for the epoch between −3 and 4.5 s, with baseline corrected as −3 to −2 s.

Next, the EEG data were used to compute event-related spectral perturbations through fast Fourier transform analysis with a three-cycle wavelet Hanning window. The computation yielded a three-axis plot containing the amplitude of each frequency component, latency time, and frequency vector [29] (Figure 4).

The following equation was used according to the method of Pfurtscheller and Aranibar to calculate ERD/ERS values for statistical analysis [30]: *ERD/ERS(k)* = $\frac{A\left(k\right)-R}{R}\times 100\%$, where *A* denotes the power at sample *k*, and *R* is the mean power at baseline. During this process, the ERD/ERS time window ranged from −3 to 4.5 s, with a baseline period ranging from −3 to −2 s. The data were divided according to three bands of interest: alpha (8–12 Hz), low-beta (13–20 Hz), and high-beta (21–30 Hz) frequency bands [31].

#### 2.2.3. Gait Analysis

Out of the 11 patients in the RAGT group, seven were eligible for gait analysis before and after RAGT. After the length of the patients’ legs was measured, they were asked to walk across the Proto Kinetics Movement Analysis Software walkway four times. Patients were required to stand up from a chair with armrests, walk 3 m, turn around, and return to the chair and sit down as quickly as possible. For each patient, three trials were recorded, and the mean time required to perform the task was calculated [32]. The mean time interval between each trial was 1 min. Gait parameters, such as walking speed, cadence, step length, stride length, stride width, and gait cycle duration, were measured and assessed with respect to their correlations with ERD and ERS. Among the parameters that assessed individual feet, the mean and difference between values for both feet were calculated for statistical analysis. Of the 11 patients in the RAGT group, four experienced difficulty walking along the path without assistance and thus withdrew from the gait analysis. Age-matched healthy controls were also assessed.

### 2.3. Statistics

For the primary clinical score, we performed a two-way repeated measures analysis of variance (ANOVA) with the within-subject factor of time (before and after rehabilitation), and a between-subject factor (traditional and RAGT). In the event that a significant interaction was identified, a paired *t* test was performed for post hoc analysis to determine the improvement resulting from rehabilitation in each group.

The peak ERD from EMG onset (between −3000 m/s to 1000 m/s) and peak ERS from EMG offset (between −250 m/s to 4500 m/s) were first detected using contralesional or ipsilesional electrodes. ERD and ERS from the ipsilesional C3 equivalent electrode and gait parameters were analyzed using a paired sample *t* test with the within-subject factor of time (pre- and post-RAGT) to show the effect of the RAGT. Additionally, independent-sample *t*-test with the between-subject factor of group (healthy controls and patients with stroke pre-RAGT) as tested to confirm the difference between participants with stroke and healthy controls.

A Pearson correlation coefficient test was also performed on all of the parameters to detect possible correlations with the clinical scales.

## 3. Results

One of the 12 participants in each group withdrew from training because of the co-morbidity of stroke; thus, 11 patients in each group completed rehabilitation. Table 1 lists the geographic data of the recruited patients. No differences were noted in age, gender, stroke type and onset duration.

### 3.1. Primary Clinical Scores

**Figure 5:**
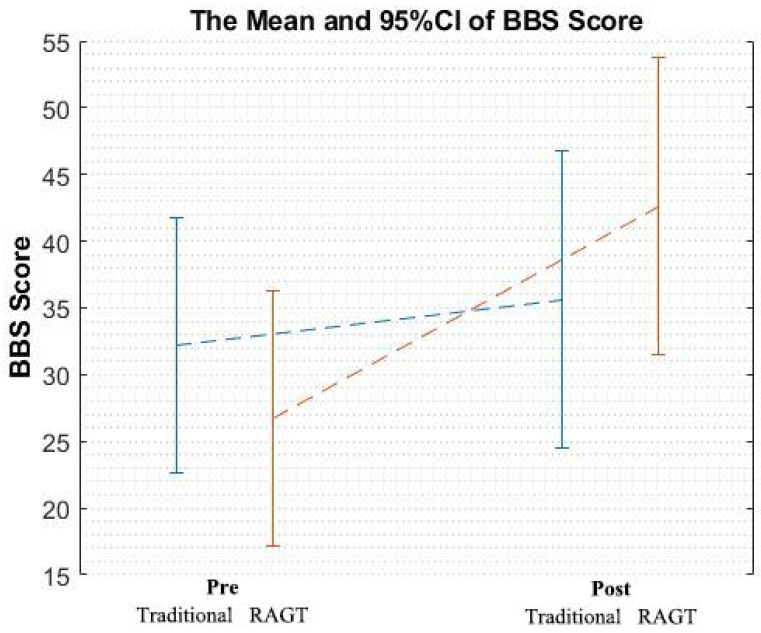
A graphical representation of the results of Berg balance scale (BBS) scores (mean and 95% confidence intervals).

The clinical scores, BBS, were also shown in Table 1. A two-way ANOVA was conducted on the improvement resulting from each rehabilitation type (RAGT and traditional rehabilitation). The interaction effect of group × time was significant (*F* (1,20) = 7.98, *p* = 0.011). A graphical representation of the results of BBS scores (mean and 95% confidence intervals) was showed in Figure 5. A post hoc analysis revealed a significant improvement in the RAGT group (*t* (10) = 4.71, *p* = 0.001) but not in the traditional treatment group (*t* (10) = 1.22, *p* = 0.252).

### 3.2. Secondary Parameters

#### 3.2.1. Gait Analysis

Additional gait analysis was performed only on patients in the RAGT group, among whom seven were capable of participation. In the walking assessment, the speed (*t* (17) = 9.80, *p* < 0.001) and cadence (*t* (17) = 8.96, *p* < 0.001) of participants in the healthy control group were significant higher than those of patients in the treatment group, and a trend of improvement in speed and cadence after RAGT was noted in the patients with stroke (*t* (6) = 1.97, *p* = 0.096; *t* (6) = 2.36, *p* = 0.056; Table 2).

As expected, after mean values were calculated for the parameters of the left and right feet, the step length (*t* (17) = 7.35, *p* < 0.001), stride length (*t* (17) = 7.41, *p* < 0.001), stride width (*t* (8.70) = 3.16, *p* = 0.012), and gait cycle duration (*t* (6.19) = 4.93, *p* = 0.002) were inferior in the patients with stroke prior to RAGT treatment compared with the healthy controls.

Post-RAGT improvements in gait cycle duration ((*t* (6) = 3.38, *p* = 0.015) parameters were noted. The differences between parameters for the left and right feet were then analyzed; the step length (*t* (6.44) = 3.81, *p* = 0.008) was generally the same for the left and right foot among participants in the control group but significantly different among participants with stroke in the pre-RAGT group. A significant difference in gait cycle duration after RAGT was also noted (*t* (6) = 2.73, *p* = 0.034).

#### 3.2.2. Changes in ERD and ERS

Additional EEG analysis was performed only on patients in the RAGT group, among whom eight were capable of participation. We also recruited 12 age-matched healthy controls to obtain age-matched ERD and ERS data. A significant difference was noted for ERS in the low-beta and high-beta frequency bands of the ipsilesional cortex between the pre- and post-RAGT measurements (*t* (7) = 2.65, *p* = 0.033; *t* (7) = 2.63, *p* = 0.034). ERD was not significantly different between the pre- and post-RAGT measurements in three frequency bands. On the other hand, ERD and ERS were not different in the healthy controls compared with the patients with stroke pre-RAGT in three frequency bands.

### 3.3. Correlations of Clinical Outcomes with ERD and ERS

**Figure 6:**
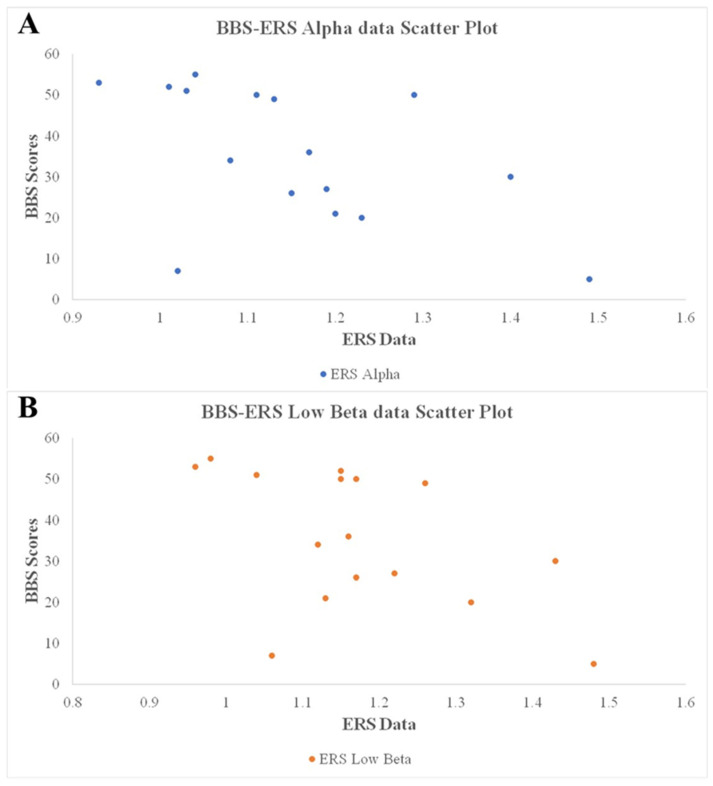
The Scatter Plot of correlations of clinical outcomes with ERS. (**A**) ERS alpha. (**B**) ERS low beta.

Pearson correlation coefficient analysis revealed strong correlations of BBS scores with gait speed (*r* = 0.91, *p* < 0.001) and cadence (*r* = 0.87, *p* < 0.001) and significant correlations of BBS scores with ipsilesional alpha and low-beta ERS (*r* = −0.52, *p* = 0.039; *r* = −0.52, *p* = 0.040). The raw data of correlations of clinical outcomes with ERD and ERS is shown in Figure 6.

## 4. Discussion

In summary, we identified significant improvements in BBS scores after additional RAGT training in primary clinical scores. In addition, in our preliminary result, an improvement of gait cycle duration and a decrease in ipsilesional low- and high-beta ERS after RAGT were also observed. We also been the first to identified negative correlations between BBS scores and ipsilesional alpha and low-beta ERS.

A BBS score of <45 indicates a potential risk of falling among older adults [33]. In the current study, the patients with stroke had an average age of approximately 60 years, and the average baseline BBS scores were 26.73 among patients in the RAGT group and 32.18 among those in the traditional treatment group (Table 1). Participants in the RAGT group exhibited an improvement of more than 7 points, which indicated a true change in balance [34]. However, the improvement in the RAGT group (average difference of 15.91) was more than fourfold greater than that in the traditional treatment group (an average difference of 3.46), which may indicate the greater clinical efficacy and added benefits of additional RAGT interventions.

Gait analysis in the present study indicated significant improvements among patients in the RAGT group. Gait cycle duration decreased significantly among patients in the RAGT group, which suggested an improvement in walking ability. Moreover, the correlation results supported the suitability of using objective biological measurements to assess stroke severity. One study suggested that balance is a significant factor that influences walking speed in patients with chronic stroke [35]. Gait analysis involves more intuitively relevant parameters such as speed and cadence, which determine the overall gait performance and observable improvement of patients with stroke [36]. Criterion-related validity has been supported by moderate to high correlations between BBS scores and other functional measurements among older adults with motor disability [10,33,37]. The BBS can be used at the time of rehabilitation admission to predict the degree of improvement in walking ability for patients with stroke [38]. BBS scores and their correlation with gait parameters in the present study reveal consistent improvements resulting from RAGT rehabilitation among patients with stroke.

The preliminary finding of decrease in ERS observed in the low-beta and high-beta frequency bands in RAGT group might be a worthwhile exploration in the future study. Although ERS has been reported to be suitable for biometric authentication [19], reflecting declines in the excitability of cortical neurons [39], weaker ERS is considered indicative of stronger neural plasticity of the brain [22]. The decrease in ERS among patients with stroke suggests that their brain plasticity is enhanced. A study concluded that beta ERS induced by somatosensory stimulation reflects aspects concerning the functional state of the primary motor cortex [25] and reported that post-stimulus beta ERS power was significantly suppressed during active movement (cube manipulation).

Of interest, significant negative correlations were observed between BBS scores and low- and high-beta bands in ipsilesional ERS. The decreasing trend in ipsilesional alpha ERS and significant difference in ipsilesional low-beta ERS between patients before and after RAGT seemed to suggest correlations of BBS scores with ipsilesional alpha and low-beta ERS. This suggests that changes in sensorimotor rhythm may reflect changes in the plasticity of the brain resulting from improvements in motor function of the lower extremities (balance).

GABA_A_ receptors mediate tonic currents that hyperpolarize thalamocortical neurons and modulate their firing pattern which is not time-locked to presynaptic action potentials [40]. It could contribute to modulating network oscillation in EEG [41]. Inhibiting tonic GABA signaling during the stroke repair phase was reported to enhance functional recovery in mice. Therefore, tonic GABA may also play an important function in modulating brain repair [42]. A speculation has been proposed that the underlying mechanism involving GABA tonic currents which enhanced functional recovery and also changed the EEG patterns in our stroke patents. Future studies in animal model should be done in order to prove this important concept.

ERD, ERS, and gait measurements in the present study demonstrated the benefit of RAGT, and we expect that the results would have indicated a more distinct improvement if our research had not been limited by a small sample size and strictness of measurement criteria.

The limitations of this study include relatively small number of stroke patients enrolled in the EEG and gait analysis. The lack of a sham RAGT control group can only provide preliminary and exploratory results in EEG and gait analysis but might be worthwhile exploration in the future study. A large number and sham controlled study should be enrolled in order to illustrate the suitability of the EEG and gait analysis as objective parameters in clinical study.

## 5. Conclusions

This study demonstrated that RAGT combined with traditional rehabilitation can improve balance and gait, as evidenced by increased BBS scores and reduced gait cycle duration following RAGT intervention. Significant reductions in low- and high-beta bands in ipsilesional ERS were observed. Additionally, correlations were noted between BBS scores and gait speed and cadence and between BBS scores and ipsilesional ERS. These findings suggest the possible relationship between brain oscillation and clinical outcomes in affecting stroke rehabilitation.

## Figures and Tables

**Table 1:** Demographics and statistics of clinical scores between participants in the traditional rehabilitation and RAGT groups before and after training.

	Stroke-Traditional	Stroke-RAGT	*p*	Statistic
*n* = 22	11	11		
Gender, *n* (%)			0.666	*t* (20) = 0.439
Male	8 (72.7)	7 (63.6)		
Female	3 (27.3)	4 (36.4)		
Age (years)	61.27 ± 9.79	61.82 ± 7.97	0.887	*t* (20) = 0.143
Type of injury			0.400	*t* (20) = 0.861
Ischemia	6 (54.5)	8 (72.7)		
Hemorrhage	5 (45.5)	3 (27.3)		
Affected Limb			0.682	*t* (20) = 0.415
Left	7 (63.6)	6 (54.5)		
Right	4 (36.4)	5 (45.5)		
Time post-stroke (month)	18.09 ± 19.58	25.36 ± 17.17	0.365	*t* (20) = 0.926
BBS Score				
Pre-rehabilitation	32.18 ± 15.14	26.73 ± 15.38	0.011 **	*F* (1,20) = 7.97
Post-rehabilitation	35.64 ± 22.11	42.64 ± 11.99		

** *p* < 0.05.

**Table 2:** Values and statistics of ERD, ERS, and gait analysis between healthy controls and patients with stroke before and after RAGT.

	Healthy Control	Stroke Pre	Stroke Post	Control-Pre *p*-Value	Pre-Post *p*-Value
Gender	7M5F	7M5F		N/A	N/A
Age	61.25 ± 6.75	62.83 ± 6.88		N/A	N/A
ERD Ipsilesion					
Alpha	0.64 ± 0.14	0.73 ± 0.21	0.74 ± 0.20	0.314	0.630
Low Beta	0.64 ± 0.14	0.74 ± 0.18	0.74 ± 0.20	0.179	0.541
High Beta	0.67 ± 0.14	0.77 ± 0.16	0.78 ± 0.18	0.165	0.804
ERS Ipsilesion					
Alpha	1.14 ± 0.182	1.18 ± 0.19	1.12 ± 0.096	0.647	0.054
Low Beta	1.20 ± 0.205	1.23 ± 0.18	1.12 ± 0.069	0.730	0.033 **
High Beta	1.23 ± 0.179	1.26 ± 0.17	1.11 ± 0.097	0.779	0.034 **
GAIT analysis					
Walking speed (cm/s)	101.29 ± 15.15	26.61 ± 15.17	35.52 ± 15.18	0.000 **	0.096
Walking cadence (steps/min)	108.81 ± 8.42	61.13 ± 13.51	72.56 ± 18.84	0.000 **	0.056
Step Length Mean (cm)	55.36 ± 8.03	24.45 ± 10.18	27.65 ± 9.30	0.000 **	0.195
Step Length Sub (cm)	1.75 ± 1.68	11.65 ± 6.76	11.59 ± 6.54	0.008 **	0.977
Stride Length Mean (cm)	110.93 ± 15.87	48.85 ± 20.42	55.31 ± 18.92	0.000 **	0.190
Stride Length Sub (cm)	1.21 ± 1.40	0.471 ± 0.352	0.772 ± 0.764	0.108	0.294
Stride Width Mean (cm)	11.74 ± 2.19	16.44 ± 3.57	17.60 ± 3.98	0.012 **	0.322
Stride Width Sub (cm)	0.377 ± 0.399	0.113 ± 0.133	0.136 ± 0.087	0.111	0.747
Gait Cycle Dur Mean (s)	1.10 ± 0.084	2.05 ± 0.507	1.74 ± 0.539	0.002 **	0.015 **
Gait Cycle Dur Sub (s)	0.018 ± 0.020	0.041 ± 0.053	0.029 ± 0.056	0.181	0.034 **

** *p* < 0.05.
